# Interim Report of the Cave Committee

**Published:** 1921-03-26

**Authors:** 


The Hospital

The Workers' Newspaper of Administrative Medicine and Institutional Life. Administration. National Insurance and Health.

No. 1316, Vol. LXIX. SATURDAY, MARCH 26, 1921. PRICE TWOPENCE

### INTERIM REPORT OF THE CAVE COMMITTEE.

**"The evidence already received has convinced us that it is desirable in the public interest to maintain the voluntary system of hospital management."**

Although, as announced in the House of Commons, Viscount Cave's Committee on the financial position of voluntary hospitals will continue to hear evidence until the beginning of May, and the full report will not be published until late in that month, the Committee wish to express an opinion on an urgent matter, and in so doing preface their findings by the momentous words which stand at the head of this column. As indicated by the public announcements that have been made, as far as individual hospitals are concerned witnesses from the hospitals of London only have yet been heard, and those from provincial centres have still to offer evidence. The Committee having, however, had the advantage of the widely-gathered knowledge in the possession of such bodies as the British Red Cross Society and the British Hospitals Association, evidently consider that they are in a position to express an opinion on the vital question of State or voluntary management. That they have been able to do so in such unequivocal terms will go far to hearten the hospitals of the country to new efforts in their aim for perfection, to loosen the purse-strings of many who are in doubt as to their moral responsibilities with regard to these institutions, and to quiet those who would nationalise every conceivable institution and industry, regardless of cost and inconvenience to the community.

To the many thousands of workers for hospitals throughout the land who have through bad and good times striven to uphold and strengthen one of the most precious heritages of the race the moment is a proud and solemn one, and marks an epoch in the history of healing.

Having touched upon the most important feature of the interim report, the full text of which will be found on page 597, we must pass to the matter of detail which is the immediate cause of the early issue of this important pronouncement of the Committee of Inquiry. That feature is the position of voluntary hospitals as regards the treatment of patients insured under the National Health Acts. The Committee has appreciated that this point is urgent, having regard to the fact that many approved societies are now engaged in framing schemes with a view to dispose of the huge accumulations of money which for one reason and another remain in their hands after all of the statutory benefits have been provided for. We are glad, for reasons which will ensue, that the Committee has been at some pains to explain how these large sums, which it is thought will amount to over £7,000,000, have accrued. To a material extent the result is attributed to conditions arising out of the war, such as the reduction, owing to war conditions, of sickness, disablement, and maternity claims, and the release of reserves in respect of members killed on active service. That the accumulations are also in a measure due to hospital treatment which relieved insured patients from suffering, reducing the periods during which they are a charge on the insurance funds, is a finding of the Committee and a distinct point in the position which has occasioned the issue of the interim report.

But it is very necessary for everybody concerned to understand that what has arisen out of the present quinquennial valuation is not likely to be repeated at the end of the next period. That is to say that the conditions which have brought about the large surplus are not recurrent conditions and that financial prudence demands that the whole question of the disposal of funds must be examined with the utmost circumspection. If, as has been suggested in some quarters, the hospitals take a rapacious attitude, which nothing will satisfy but an immediate handing over of a large part of the seven millions to satisfy past claims, they would put themselves in the wrong with a great insurance system which, despite its many faults, is associated with the interests of a considerable section of the community. If, on the other hand, the societies are advised to take advantage of their present position to provide a reserve, if they can do so with present or amended powers, to afford an adequate hospital benefit for their members, commencing without delay, another and we think a wiser course of action is open. This we think is in the minds of the Committee when it is recommended that the Ministry of Health shall make any necessary amendment in the regulation bearing upon the point, shall forthwith bring this matter to the notice of the societies concerned, and advise them as to the amount of the contributions which might reasonably be made out of the available surplus, and shall not approve any scheme for the disposal of a surplus until the suggestion has received due consideration.

We consider, then, that there is little occasion for ill-considered utterances such as "the coming clash between the hospitals and the Government," and warnings to the hospitals as to the acceptance of "dangerous gifts." We do not think that the outcome, whichever way it is regarded, can conceivably have within it danger of public control, whatever way the surpluses are applied to liquidate the just obligations of the societies. The Ministry of Health has its hands fairly full for some time to come, and we are entirely confident that Dr. Addison is literally sincere in his expressed wish to uphold the voluntary system without any State interference. The suggestion that the approved societies, even could the Acts of Parliament, Charters, Board of Trade Sanctions, or other form of constitution governing the hospitals be altered for the purpose, should demand seats on Boards of Management for their nominees, because they have been parties to a clear business transaction, is not easy to believe. We regard the position as one which does not present any probable complications, and, from the point of view of the hospitals, as well as the approved societies and their members, one which presents every cause for congratulation.

